# An integrated model of acinar to ductal metaplasia-related N7-methyladenosine regulators predicts prognosis and immunotherapy in pancreatic carcinoma based on digital spatial profiling

**DOI:** 10.3389/fimmu.2022.961457

**Published:** 2022-07-28

**Authors:** Hao Yang, Julia Messina-Pacheco, Andrea Liliam Gomez Corredor, Alex Gregorieff, Jun-li Liu, Ali Nehme, Hamed S. Najafabadi, Yasser Riazalhosseini, Bo Gao, Zu-hua Gao

**Affiliations:** ^1^ Department of General Surgery, The Second Affiliated Hospital of Harbin Medical University, Harbin, China; ^2^ Department of Pathology, McGill University and the Research Institute of McGill University Health Centre, Montreal, QC, Canada; ^3^ MeDic Program, The Research Institute of McGill University Health Centre, & Division of Endocrinology and Metabolism, Department of Medicine, McGill University, Montreal, QC, Canada; ^4^ Department of Human Genetics, McGill University, Montreal, QC, Canada; ^5^ McGill University Genome Centre, Montreal, QC, Canada; ^6^ Department of General Surgery, Peking University People’s Hospital, Beijing, China; ^7^ Department of Pathology and Laboratory Medicine, British Columbia (BC) Cancer Research Center, University of British Columbia, Vancouver, BC, Canada

**Keywords:** acinar to ductal metaplasia, N7-methyladenosine, pancreatic carcinoma, prognosis, immunotherapy, digital spatial profiling

## Abstract

Acinar-to-ductal metaplasia (ADM) is a recently recognized, yet less well-studied, precursor lesion of pancreatic ductal adenocarcinoma (PDAC) developed in the setting of chronic pancreatitis. Through digital spatial mRNA profiling, we compared ADM and adjacent PDAC tissues from patient samples to unveil the bridging genes during the malignant transformation of pancreatitis. By comparing the bridging genes with the 7-methylguanosine (m7G)-seq dataset, we screened 19 m7G methylation genes for a subsequent large sample analysis. We constructed the “m7G score” model based on the RNA-seq data for pancreatic cancer in The Cancer Genome Atlas (TCGA) database and The Gene Expression Omnibus (GEO) database. Tumors with a high m7G score were characterized by increased immune cell infiltration, increased genomic instability, higher response rate to combined immune checkpoint inhibitors (ICIs), and overall poor survival. These findings indicate that the m7G score is associated with tumor invasiveness, immune cell infiltration, ICI treatment response, and overall patients’ survival. We also identified FN1 and ITGB1 as core genes in the m7Gscore model, which affect immune cell infiltration and genomic instability not only in pancreatic cancer but also in pan-cancer. FN1 and ITGB1 can inhibit immune T cell activition by upregulation of macrophages and neutrophils, thereby leading to immune escape of pancreatic cancer cells and reducing the response rate of ICI treatment.

## Introduction

Pancreatic ductal adenocarcinoma (PDAC) is the most important histological subtype of pancreatic cancer, accounting for approximately 90% of all pancreatic cancers. The 5-year survival rate of PDAC is less than 5%, and the median survival time after diagnosis is less than 6 months (1). The poor prognosis of PDAC has been attributed to multiple factors including late diagnosis, the lack of sensitive and specific biomarkers to detect PDAC, and the lack of effective measures to prevent its development and interrupt its progression (2). Studies have shown that in pancreatitis, pancreatic acinar cells lose their morphology and characteristics, undergo cell transdifferentiation and acquire ductal morphology and characteristics. This process is called acinar to ductal metaplasia (ADM) (3). ADM developed in the setting of acute pancreatitis is usually transient and reversible. However, persistent ADM in the setting of chronic or recurrent pancreatitis may progress to pancreatic intraepithelial neoplasias (PanIN) and eventually to invasive tumor (4, 5) (6, 7). Studies have found that when ADM occurs, a variety of signaling pathways in acinar cells are activated (Notch, Wnt, PI3K/AKT, etc.), which inhibits the transcription of specific genes in acinar cells (e.g. *Mist1, Cpa1, Amy2a*, etc), while duct cell genes (e,g, *Krt19, Sox9*, etc.) are upregulated (8, 9). Previous reports have demonstrated that suppression of transdifferentiation signals in these cells blocks subsequent PanIN and PDAC (10). Therefore, elucidating the key bridging molecules in the malignant process of ADM-related PDAC can not only help us find a novel mechanisms of PDAC pathogenesis, but also provide us with new therapeutic and preventive strategies against PDAC.

Epitranscriptomics provides insights into the biological and pathological roles of different RNA modifications. An emerging type of RNA methylation, 7-methylguanosine (m7G) modification, has been a research hotspot over the past two years. Studies have shown that m7G modification is one of the most common forms of base modification in post-transcriptional regulation (11), and is widely distributed in the 5’ cap region of tRNA, rRNA, and eukaryotic mRNA (12). m7G methylation was found to play an important role in the development of a variety of cancers, including colon and lung cancer (13, 14). m7G-related epigenetic regulation can also affect the tumor immune microenvironment and the efficacy of immunotherapy (15). The m7g modification process is regulated by a collection of key genes including *mettl1, mettl3*, *Cdk1*, etc. (14, 16) However, the role of m7g modification and its underlying regulatory genes in the malignant progression of PDAC is still unclear.

In this study, we compared ADM and adjacent PDAC tissues from pancreatic cancer patients and identified high and low-expressed bridging genes during the malignant transformation of pancreatitis through digital spatial mRNA profiling (DSP) (17). There was a high degree of overlap between these bridging genes and the m7G methylation genes. After comparing the bridging genes with the m7G-seq dataset, we selected 21 m7G methylation genes for subsequent bioinformatics analysis. Based on these 21 m7G methylated genes, we constructed a model, the m7Gscore, and used it to classify potential molecules that are associated with different patterns of immune infiltration and genomic instability in PDAC. We also evaluated whether m7G score and m7G target genes could be used to predict patients’ response to immune checkpoint inhibitors (ICIs). As the core genes of m7G score model, FN1 and ITGB1 are highly expressed not only in the stroma and epithelial cells of ADM and PDAC, but also in pan-cancer. FN1 and ITGB1 also affect Overall survival rate, immune cell infiltration, tumor mutation burden and microsatellite instability in pan-cancer. Finally, we concluded that FN1 and ITGB1 can also up-regulate macrophages and neutrophils and inhibit immune T cell activition in pancreatic cancer, leading to immune escape and reducing the response rate of ICIs treatment.

## Materials and methods

### Sample collection

**Figure 1:**
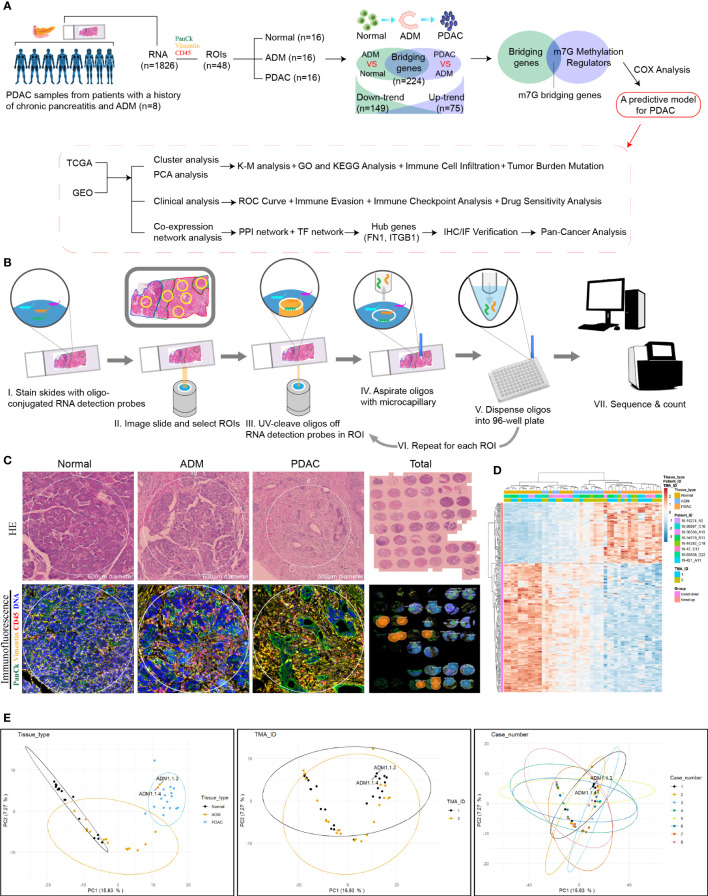
Digital spatial profiling of pancreatic cancer FFPE samples from patients with a history of chronic pancreatitis and ADM. **(A)** Flowchart of the study. **(B)** Schematic overview of the DSP workflow. **(C)** Representative HE staining and IHC images from each group. **(D)** Heatmap of the 1826 detected genes. Cluster analysis indicated the clusters marked in different colors. **(E)** PCA analysis of tissues, slides and patients.

The experimental design and analysis are shown in the flow chart (**Figure 1A**). With the approval of the institutional ethics review board of the McGill University Health Center, a total of 8 sets of PDAC tissue samples were obtained from 8 patients with a history of chronic pancreatitis who underwent surgical resection in McGill University Health Centre. In each case, formalin fixed paraffin embedded (FFPE) tissue blocks that contain normal acini, ADM tissue and PDAC on the same tissue section were selected. The clinical features of the eight patients are shown in **Table 1**. None of the patients with pancreatic cancer received any pre-operative treatments, including radiotherapy, chemotherapy, or biological treatments. All specimens were histopathologically diagnosed by two pathologists according to the WHO diagnostic criteria for PDAC (18).

**Table 1:** Patient characteristics.

CASE No.	Age	Sex	Grade	Stage
CASE 1	67	M	G2	pT3N1
CASE 2	80	M	G2	pT3N1
CASE 3	86	M	G2	pT2N0
CASE 4	66	F	G2	pT2N0
CASE 5	83	M	G2	pT2N1
CASE 6	67	F	G1	pT1cN0
CASE 7	83	F	G1-G3	pT3N1
CASE 8	66	M	G3	pT2N

### NanoString Technologies’ digital spatial mRNA profiling

We selected several regions of interest (ROIs) from each PDAC sample, including normal, ADM and PDAC ROIs. NanoString Technologies’ newly developed GeoMx™ digital spatial profiling (DSP) technology allows for morphology-driven, high-plex spatial analysis of FFPE samples (17). Using the GeoMx Cancer Transcriptome Atlas, a panel of RNA probes designed for comprehensive profiling of the tumor, tumor microenvironment, and tumor immune status with 1833 RNA targets, we directly analyzed the *in situ* RNA expression of a total of 48 ROIs from 8 PDAC samples. Briefly, RNA probes coupled to unique photocleavable oligonucleotide tags are hybridized to slide-mounted FFPE tissue sections. Slides are then stained and visualized, and the oligonucleotides are then released from ROIs *via* UV exposure. The oligonucleotides are collected separately and quantified. Counts are then mapped back to each tissue location, yielding high quality, spatially resolved differential gene expression profiles. The flowchart of DSP technology was shown in **Figure 1B**.

### Histology, immunohistochemistry and immunofluorescence

Formalin fixed tissue was processed, embedded in paraffin, and cut into 5 µm sections. Hematoxylin and Eosin (H&E) (Thermo Fisher Scientific, 7221, 7111) staining was performed according to the clinical laboratory standard. Two areas of normal acini, ADM and PDAC from each case were selected for the construction of a tissue microarray. Immunohistochemical (IHC) staining was performed using antibodies against Fibronectin 1 (FN1, 1:1000, Cell Signaling Technologies 26836) and integrin β 1(ITGB1, 1:1000, Cell Signaling Technologies 34971). Tissue sections were deparaffinized in xylene and rehydrated in graded ethanol. Antigen retrieval was performed by heating sections in boiling sodium citrate buffer (Sigma-Aldrich, C-9999) for 20 minutes. After blocking with 3% hydrogen peroxide and bovine serum albumin (BSA), the tissues were incubated with the primary antibody at 4°C overnight. After washing, the tissues were incubated with corresponding horseradish peroxidase (HRP)-conjugated secondary antibodies. The color was developed using diaminobenzidine (DAB) substrate (Sigma-Aldrich, D-7304) and slides were counterstained with hematoxylin. Images of three random areas from each section were captured at 400x, 500x, and 600x magnification for evaluation. Immunofluorescence staining was performed using primary antibodies against cytokeratin-19 (CK19; 1:500, DSHB, TROMAII), alpha-smooth muscle actin (SMA, 1:2000, Sigma-Aldrich A2547), FN1 (1:200), and ITGB1 (1:200). Corresponding Alexa Fluor dyes were used for fluorescent detection. DAPI was used for nuclear counter staining. Images were captured on the Zeiss LSM780 laser scanning confocal microscope.

### Data retrieval and processing

We obtained the m1A dataset (1655 regulator genes) and m5C dataset (34 regulator genes) through the RMBase database (https://rna.sysu.edu.cn/rmbase June 2021) (19, 20). We acquired the m6A dataset (417 regulator genes) through the M6A2Target database (https://m6a2target.canceromics.org June 2021) (21). We obtained the m7G-seq dataset (2795 regulator genes) through the m7GHub database (https://www.xjtlu.edu.cn/biologicalsciences/m7ghub June 2021) (22). We intersected each group of methylated genes with the bridging genes to identify the proportion of each methylated gene in the bridging gene set. The bridging genes and the m7G-seq dataset were intersected to obtain 54 m7G methylation genes. Through the Cancer Genome Atlas (TCGA) database (https://portal.gdc.cancer.gov/: accessed June 2021), we obtained the raw mRNA matrix data of PDAC in fragments per kilobase million (FPKM) format and the copy number data for pancreatic cancer. The raw data of the mRNA matrix were processed to remove duplicate samples. We also obtained the clinical data of pancreatic cancer patients through the TCGA database. To reduce statistical error, patients with survival times less than or equal to 90 days were excluded from the data. We downloaded the GSE21501 dataset from the Gene Expression Omnibus (GEO) database to obtain the mRNA matrix and clinical data of pancreatic cancer. The FPKM matrix of pancreatic cancer was converted to the TPM format and then merged with the GEO matrix, and some missing genes were removed through batch correction to expand the sample size for subsequent analysis. Similarly, patients with survival times less than or equal to 90 days were excluded from the GEO database. We download pan-cancer raw mRNA matrix data, clinical data and copy number data through UCSC database (Xena.ucsc.edu/December 2021). The clinicopathological characteristics of the pancreatic cancer patients in the TCGA database and the GEO database are shown in **Table 2**.

**Table 2:** Baseline characteristics of patients from TCGA and GEO database.

Clinical features	Total patients (317)		TCGA (185)		GSE21501 (132)	
	Number	Percentage (%)	Number	Percentage (%)	Number	Percentage (%)
Fustat						
Alive	121	38.2	85	45.9	36	27.3
Dead	166	52.3	100	54.1	66	50.0
Unknown	30	9.5	0	0	30	22.7
Stage T						
1-2	49	15.5	31	16.7	18	13.6
3-4	232	73.2	152	82.2	80	60.6
Unknown	36	11.3	2	1.1	34	25.8
Stage N						
N0	78	24.6	50	27.0	28	21.2
N1	203	64.1	130	70.3	73	55.3
Unknown	36	11.3	5	2.7	31	23.5

### Cluster analysis

To investigate whether m7G methylation gene expression is associated with pancreatic cancer, we used the “ConsensusClusterPlus” package in R to classify pancreatic cancer data. When the clustering index k is between 2 and 9, k=3 is determined as the optimal number of subtypes. When k=3, the intergroup correlation is weak, while the intragroup correlation is strong. We calculated the survival curves of different clusters of pancreatic cancer using the Kaplan–Meier method and plotted them using the “survminer” package. The relationship between the expression of the m7G gene according to the pancreatic cancer classification and the clinical data of patients was shown with a heat map. The DEGs in the three clusters of PDAC were identified by using the VennDiagram package R language. Then intersected the DEGs, we obtained 907 intergenes. Using the single-sample gene set enrichment analysis (ssGSEA) algorithm, we obtained the scores of immune cells in different clusters of pancreatic cancers, and the scores were plotted as box plots using the “GSEABase” and “GSVA” packages in R. The Gene Ontology (GO) and Kyoto Encyclopedia of Genes and Genomes (KEGG) pathway files were downloaded from the GSEA website (https://www.gsea-msigdb.org June 2021). Then, the enriched functional pathways in the pancreatic cancer classifications were plotted into a heat map using the “GSEABase” and “GSVA” packages in R.

### Principal component analysis

We obtained the principal component analysis (PCA) scores for intergenes in the three m7G clusters of PDAC. PCA maps of different clusters of pancreatic cancer were then plotted using the “limma” and “ggplot2” packages in R. The m7G score of each sample was obtained *via* a PCA analysis of DEGs in the three m7G clusters (23). Based on the m7G score, we divided all the pancreatic cancer samples into high and low m7G score groups. A Sankey diagram was used to show the relationship among the three clusters of pancreatic cancer, the two m7G score types of pancreatic cancer and the overall survival rate. We analyzed the correlation between m7G scores and immune cells by using the ssGSEA algorithm. We analyzed the relationship between m7G clusters, m7G score, and tumor mutation load using the “ggpubr” and “reshape2” packages in R. Using the “limma” package, the expression levels of UBQLN4 in the high and low m7G score groups were displayed using box plots. We also used the “survival” and “survminer” packages to analyze the combined survival rate of the high tumor mutation burden group, the low tumor mutation burden group, the high m7G score group, and the low m7G score group. Through the “maftools” package, we calculated the gene frequencies of the high and low m7G score groups, and selected the top 20 genes with the highest mutation frequency to draw a waterfall chart. The “plyr” and “ggpubr” packages were used to plot different clinical traits in the high and low m7G score groups as histograms and box plots. TCGA database data is used to draw NOMO diagram and ROC curve by using “rms” package, “regplot” package and “timeROC” R packages. The time gradient of ROC was 1,2 and 3 year. TIDE signature was an algorithm for calculating T cell dysfunction and rejection in various tumors. TIDE score was not only consistent with immune escape characteristics, but also can predict the effect of immune checkpoint treatment in patients with tumor (24). We downloaded pancreatic cancer-related TIDE score, Exclusion score and Dysfunction score data from TIDE database (http://tide.dfci.arvard.edu/ June 2021). We then analyzed the difference of the scores between the high- and low- m7G score groups.

The immune checkpoint treatment scoring data for pancreatic cancer were downloaded from The Cancer Immunome Database (TCIA) (https://tcia.at/ June 2021). We then analyzed the treatment of ctla4 and pd1 immune checkpoints in pancreatic cancer according to m7G scores.

### Protein-protein interaction (PPI) networks and transcription factor regulatory networks

Through the STRING website (https://string-db.org/cgi/input.pl June 2021), the protein-protein interaction networks of the m7G methylation genes were constructed. The TSV format files of the m7G PPI networks were also downloaded. Based on the TSV files, we plotted histograms to visualize the core genes of the m7G PPI networks. From the DAVID website (https://david.ncifcrf.gov June 2021), we obtained the transcription factors associated with m7G methylation. We constructed a transcription factor regulatory networks map of the m7G methylation genes using Cytoscape software.

### Statistical analysis

The copy number variation frequency of the m7G methylation gene was obtained by calculating the increases and reductions in the number of gene copies in the TCGA samples. The “RCircos” package in R was used to plot the circle diagram of gene copy number. Cox analysis and coexpression analysis were used to map the prognostic gene network.

## Results

### Bridging the gap between ADM and PDAC: Bridging genes identified by DSP

The experimental design and analysis are shown in **Figure 1A**. In order to identify the genes that bridge the gap between ADM and PDAC, we collected 8 samples from PDAC patients and selected 6 regions of interest (ROIs) from each sample, including 2 normal, 2 ADM and 2 PDAC ROIs. The results of hematoxylin and Eosin(H&E) staining and immunohistochemistry (IHC) showed that the selection of the ROIs of Normal, ADM and PDAC were correct. Microscopic examination of the PDAC samples showed histological evidence of conversion from normal pancreatic tissue to ADM and PDAC (**Figure 1C**). GeoMx™ digital spatial profiling (DSP) analysis of paired ADM and PDAC tissues (ADM vs Normal ∩ PDAC vs ADM) identified a total of 224 trend genes, among which 75 genes showed gradually increasing expression, and 149 genes showed gradually decreased expression. The gene expression heatmap is shown in **Figure 1D**. ADM samples 1.1.3 and 1.1.4 appeared in the cluster of PDAC samples, so they were removed from the study queue (**Figure 1E**). There was no confounding effect between the remaining patients and samples.

### High expression of m7G methylation genes in pancreatic cancer

**Figure 2:**
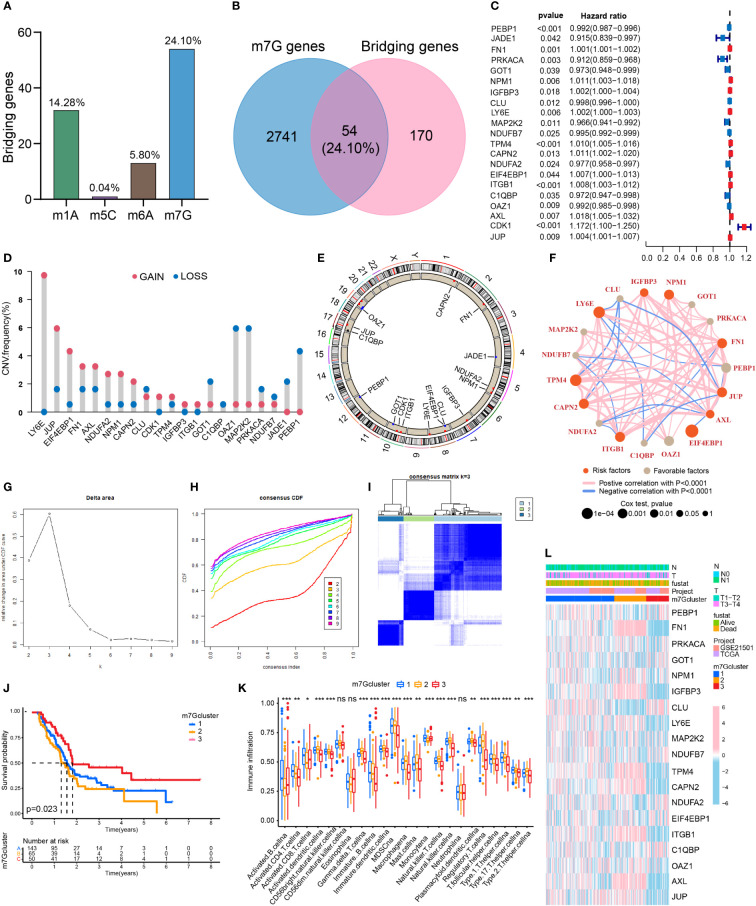
Classification of PDAC based on m7G methylation related bridging genes. **(A)** The proportion of four common methylation type genes in bridging genes. m7G methylation genes have the highest proportion. **(B)** Intersection of the bridging genes and the m7G methylation genes. **(C)** Univariate Cox analysis of the m7G methylation genes. **(D)** Diagram of the frequency of copy number variations in the m7G methylation genes. **(E)** Copy number circle diagram for the m7G methylation genes. **(F)** Prognostic network of the m7G methylation genes. **(G–I)** Classification of pancreatic cancers based on m7G methylation related bridging genes. Changes in the length and inclination of the CDF curve for k=2–9. Area under the cumulative distribution function curve for k=2–9. Division of the pancreatic cancer samples into three clusters. **(J)** Kaplan–Meier survival curves of the three clusters. **(K)** Immune cell infiltration of the three clusters. *p<0.05; **p<0.01; ***p<0.001; ns, no significance. **(L)** Heat map of pancreatic cancer classification, m7G methylation genes, and clinicopathological characteristics.

The proportions of m1A, m5C, m6A and m7G related regulators in bridging genes were 14.28%, 0.04%, 5.80% and 24.10%, respectively. Since m7G-related regulators account for a high proportion of bridging genes, we conducted more in-depth study on this type of methylation in PDAC (**Figure 2A**). The intersection of the 224 bridging genes and the 2795 m7G methylation genes yielded 54 bridging m7G methylation genes expressed in pancreatic cancer (**Figure 2B**). The 54 bridging m7G methylation genes in the TCGA dataset were subjected to univariate Cox analysis, and 21 prognostic-related bridging m7G genes were obtained (**Figure 2C**). The frequency of copy number variations in the 21 m7G methylation genes was observed with a histogram (**Figure 2D**). In most of the bridging m7G methylation genes, the frequency of copy number increases was higher than the frequency of deletions. Among these genes, the LY6E methylation gene had the most significant frequency of copy number increases. The MAP2K2 and OAZ1 methylation genes had the most significant frequency of copy number deletion. The gene copy number circle diagram shows that the m7G methylation genes were mainly concentrated on human chromosomes 8, 10, and 19 (**Figure 2E**). After merging the TCGA and GEO datasets, we obtained a total of 19 m7G methylation genes by removing some of the missing genes through batch correction. A co-expression analysis of the m7G methylation genes was performed, and a prognostic network was plotted (**Figure 2F**). As illustrated in the figure, the vast majority of the m7G genes regulate one another and form a functional ensemble that jointly affects the progression of pancreatic cancer.

### Three clusters of PDAC based on bridging m7G methylation genes

Through a cluster analysis of the bridging m7G methylation genes, we divided all the samples into three clusters (**Figures 2G–I**). The survival analysis of the three clusters of pancreatic cancer showed that cluster 2 had the lowest overall survival rate, while cluster 3 had the highest survival rate (**Figure 2J**). This indicates that the degree of malignancy of cluster 2 was relatively high, and the degree of malignancy of cluster 3 was relatively low. On the heat map (**Figure 2L**), the expression of most m7G methylation genes was significantly increased in cluster 2 and significantly decreased in cluster 3. **Figure 4K** shows that of the 23 types of immune cells, there were statistically significant differences in the expression of 20 types of immune cells among the three clusters of pancreatic cancer. These results indicate that bridging m7G methylation genes can regulate immune cell infiltration in pancreatic cancer. We performed GO and KEGG enrichment analyses between three clusters of pancreatic cancer (**Figures S1A–F**). Cluster 2 has the highest degree of malignancy, and its enrichment pathways are mainly concentrated in cell differentiation pathways, tumor microenvironment pathways and carcinogenic pathways, including regulation of cell morphogenesis involved in differentiation, ECM receptor interaction, pancreatic cancer, P53 signaling pathway and so on. The degree of malignancy of cluster 1 is weaker than that of cluster 2, and its pathways mainly focus on metabolism and tumor-related pathways, such as integrin mediated signaling pathway, movement in host environment and pathways in cancer. Cluster 3 has the lowest degree of malignancy. Moreover, 907 genes (intergenes) were overlapped among the three clusters of pancreatic cancer (**Figure S1G**). GO and KEGG analysis results showed that the intergenes were mainly enriched in ECM-receptor interaction, focal adhesion and Wnt signaling pathway (**Figures S1H–K**).

### Risk stratification of PDAC based on PCA analysis and m7G scores

**Figure 3:**
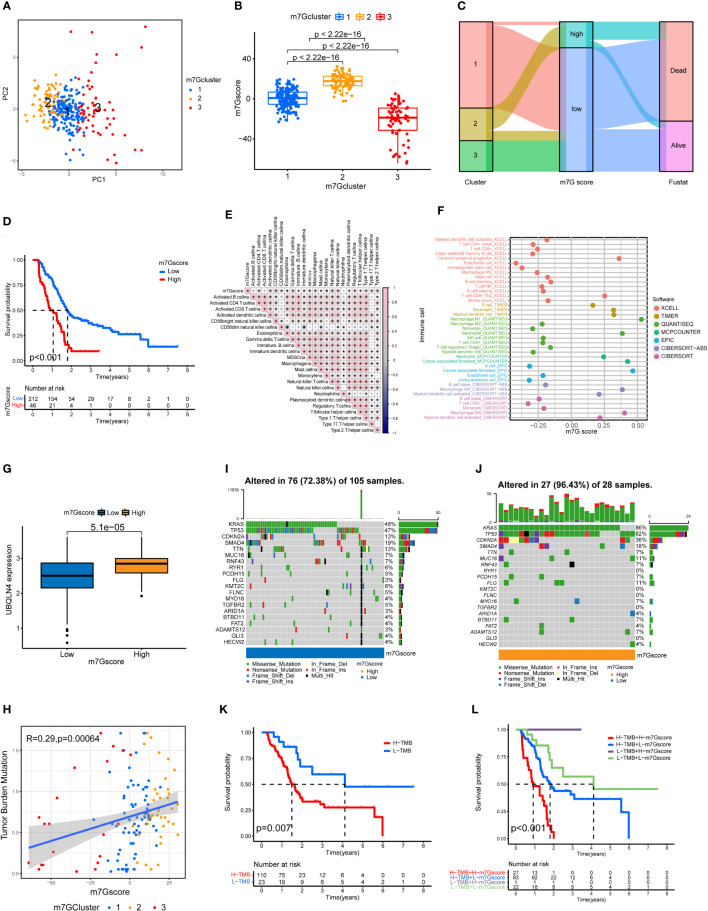
Immune cell infiltration and genomic instability in the m7G score. **(A)** PCA of the m7G methylation genes. **(B)** The m7G scores for the three clusters of pancreatic cancers. **(C)** The Sankey plots revealed the correlation results of m7G clusters, m7G scores and the future state of patients. **(D)** Kaplan–Meier survival curves of the high m7G score group and the low m7G score group. **(E)** Spearman correlation analysis of the relationship between m7G scores and immune cell types *p<0.05. **(F)** Correlation of m7G score and immune cell infiltration through multiple software. **(G)** UBQLN4 expression in the high m7G score group and the low m7G score group. **(H)** Correlation analysis of the relationship between m7G scores and tumor mutation burden among the three clusters of PDAC. **(I, J)** Waterfall plot of gene mutation frequencies for low and high m7G score groups. **(K)** Kaplan–Meier survival curves of the high tumor mutation burden group and the low tumor mutation burden group. **(L)** Combined survival analysis results for the high and low tumor mutation burden groups and the high and low m7G score groups.

Through the PCA analysis of all the samples (**Figure 3A**), we found that there was basically no overlap in m7G scores among the three clusters of pancreatic cancer, and there was a good correlation within the clusters. This indicates that our m7G classification is very accurate. **Figure 3B** shows that the m7G score was the highest for cluster 2 and lowest for cluster 3. In addition, the m7G scores of the three clusters were significantly different. Through the PCA analysis, we obtained m7G scores and divided all the pancreatic cancer samples into high and low m7G score groups. We conducted ROC analysis on the m7Gscore model and found that its 1, 2, and 3-year AUC areas were all greater than 0.6 (**Figure S1L**). Sankey diagram showed that most of the cases in cluster 2 with the highest degree of malignancy belong to the high m7G score group, most of the cases in cluster 1 belong to the low m7G score group, and all the cases in cluster 3 with the lowest degree of malignancy belong to the low m7G score group (**Figure 3C**). Moreover, the survival rate of the high m7G score group was significantly lower than that of the low m7G score group (**Figure 3D**). This indicates that a high m7G score is associated with high risk, while a low m7G score often reflects a low risk. This is consistent with the results of previous studies. Cluster 2 pancreatic cancer has a high m7G score and low survival rate, while cluster 3 has a low m7G score and a high survival rate.

### m7G score is associated with immune cell infiltration and tumor mutation burden

We performed a correlation analysis for m7G score and immune cell infiltration (**Figure 3E**). Among the 23 types of immune cells, the expression of 20 types of immune cells was statistically significantly associated with the m7G score: 18 types were positively correlated with the m7G score, and 2 types were negatively correlated with the m7G score. We also analyzed the relationship between m7G score and immune cell infiltration through multiple softwares. Multiple software results showed that m7G score was closely related to a variety of inflammatory related immune cells, including macrophage, neutrophil and cancer associated fibroblast (**Figure 3F**). **Figure 3G** shows that the expression level of UBQLN4 in the high m7G score group was significantly higher than that in the low m7G score group, indicating that the higher the m7G score was, the greater the genomic instability (25). **Figure 3H** shows that the m7G score was positively correlated with tumor mutation burden, with cluster 2 having the highest tumor mutation burden and cluster 3 having the lowest tumor mutation burden. The waterfall chart shows that the gene mutation frequency of the high m7G score group was significantly higher than that of the low m7G score group (**Figure 3I**, **J**). The genes with the highest mutation frequency in the high and low m7G score groups were KRAS, TP53, CDKN2A and SMAD4 (26, 27). **Figure 3K** shows that the survival rate of the high tumor mutation burden group was significantly higher than that of the low tumor mutation burden group. We also performed a joint analysis of the high and low tumor mutation burden groups and the high and low m7G score groups (**Figure 3L**), and the results showed significant differences (p<0.001), indicating that both the tumor mutation burden and m7G scores were correlated with patient prognosis.

### m7G score is associated the clinical behavior of PDAC

**Figure 4:**
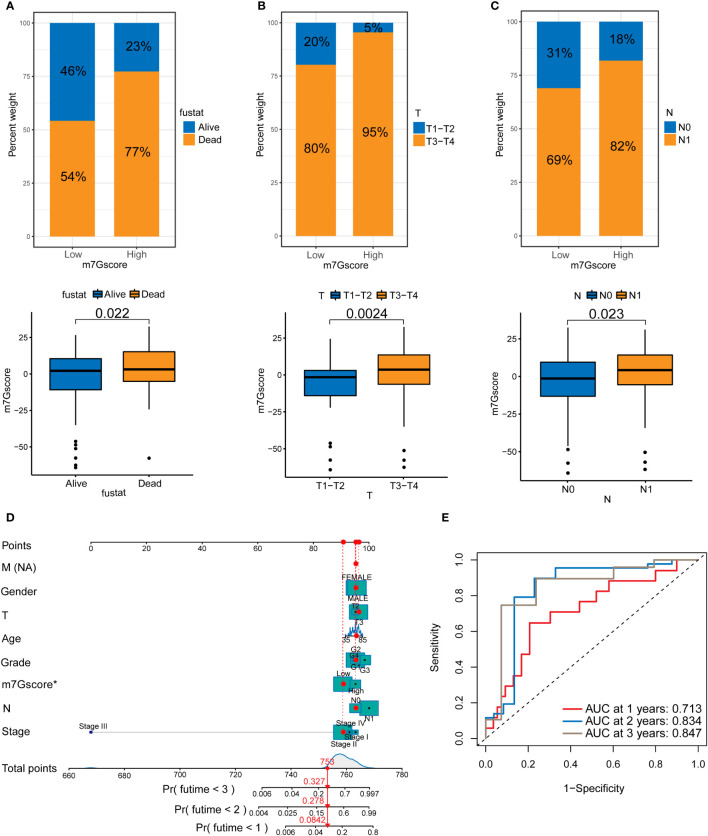
The relationship between m7G scores and prognosis in PDAC. **(A)** Survival in the high and low m7G score groups. **(B)** T staging of the high and low m7G score groups. **(C)** N staging of the high and low m7G score groups. **(D)** NOMO map associated with m7G score and clinical information. **(E)** Area under the curve (AUC) for time-dependent receiver operating characteristic curves demonstrating the prognostic performance of the NOMO model.

We analyzed the patients’ m7G scores and clinicopathological characteristics, including survival, T stage, and N stage. **Figures 4A**–**C** shows that a high m7G score was closely associated with a poor prognosis, local tumor invasion, and lymph node metastasis. This indicates that from the perspective of clinical pathological characteristics, a high m7G score represents a higher degree of malignancy. We scored various clinicopathological features and m7G scores and drew a NOMO map to predict the prognosis of patients. If the total score reaches 753, the probability that the patient’s survival time was less than 1, 2, and 3 years is 0.0842%, 0.278%, and 0.327%, respectively (**Figure 4D**). Among multiple indicators, only the m7G score was statistically significant. The ROC curve results showed that the 1, 2, and 3-year AUC areas of the NOMO model were 0.713, 0.834, and 0.847, respectively (**Figure 4E**).

### m7G score predicts tumor response to immune checkpoint inhibitor treatment

**Figure 5:**
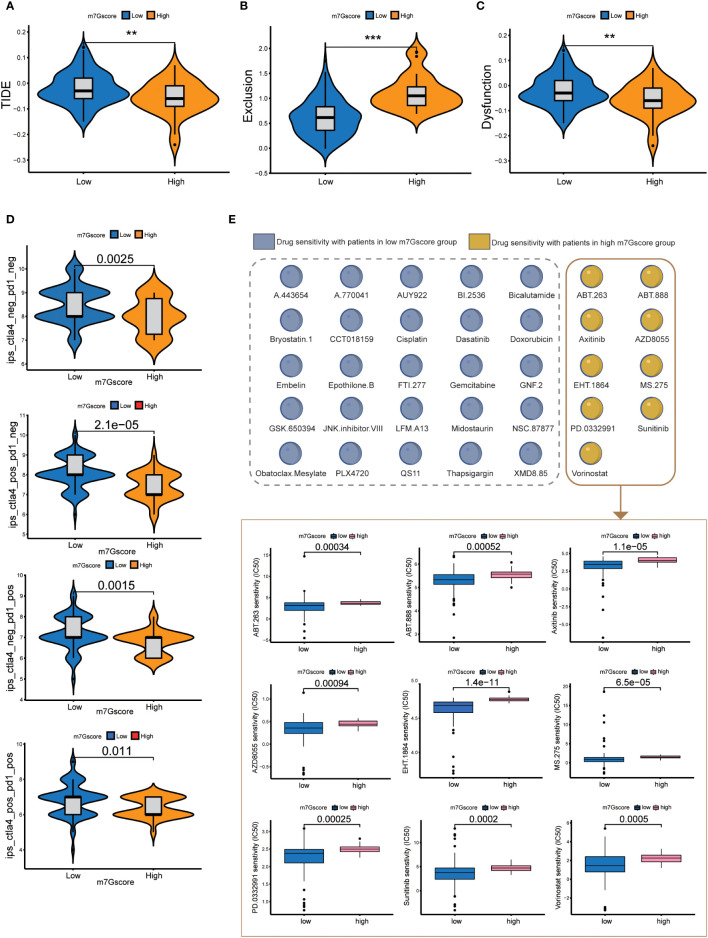
Immune checkpoint treatment and drug sensitivity. **(A–C)** TIDE score, Exclusion score and Dysfunction score of high and low m7G score groups. **p<0.01; ***p<0.001 **(D)** Immune checkpoint treatment in the high and low m7G score groups. **(E)** Drug sensitivity in the high and low m7G score groups.

The results of TIDE signatures showed that the immune evasion mechanism of the high m7G score group was mainly immune rejection, and the immune evasion mechanism of the low m7G score group was mainly immune dysfunction. The TIDE score of the low m7G score group was significantly higher than that of the high m7G score group. (**Figures 5A**–**C**) The results of immunosuppressant monotherapy and combination therapy analysis showed that tumors with low m7G scores had a higher rate of response to single immune checkpoint treatment (ICI). Although the efficacy of PD-1 and CTLA-4 as single immune checkpoint treatment was lower in patients with high m7G scores than in those with low m7G scores, the efficacy of two-drug combination immune checkpoint therapy in patients with high m7G scores was higher than that of the low m7G score group (**Figure 5D**). The observation was in agreement with the results of most drug clinical trials, in which combination immune checkpoint therapy had better efficacy than monotherapy for advanced pancreatic cancer. We also conducted drug sensitivity analysis in patients with high and low m7G score groups, so as to predict potential effective drugs for pancreatic cancer patients (**Figure 5E**). A total of 9 drugs were screened for patients with high m7G score, and 25 drugs were selected for patients with low m7G score (**Figure S2**).

### FN1 and ITGB1 were the core genes regulating m7G methylation

**Figure 6:**
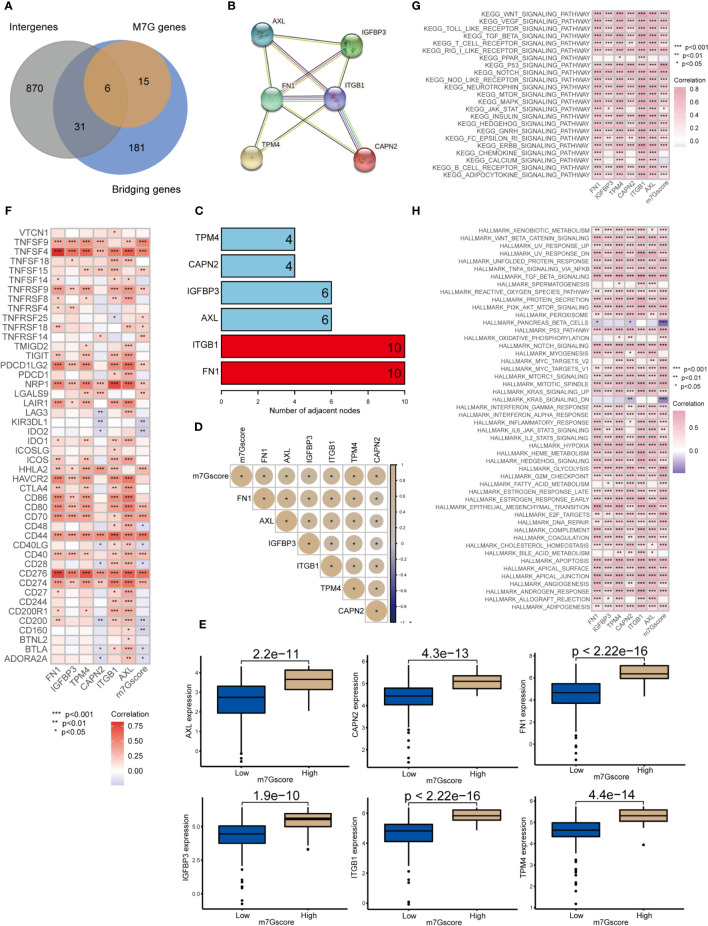
Network of m7G target genes in the m7G score model *p<0.05; **p<0.01; ***p<0.001. **(A)** Venn diagram of cluster intergenes, bridging genes and m7G target genes. **(B)** Core genes of the PPI network of m7G methylation genes. **(C)** PPI network of the m7G methylation genes. **(D)** Correlation analysis of m7Gscore and m7G target gene. **(E)** Expression of m7G target genes in high and low m7G groups. **(F)** Correlation analysis of m7G target genes and m7G score with immune checkpoint related genes. **(G, H)** GSVA analysis of m7G target genes and m7Gscore.

We obtained 6 m7G target genes by taking the overlapping genes between clusters intergenes, m7G genes, and bridging genes (**Figure 6A**). By constructing a PPI network (**Figures 6B, C**), we found that the m7G methylation genes were closely related to each other and could interact with one another to form a functional ensemble and jointly regulate the occurrence and development of tumors. Among these, FN1 and ITGB1 were the core genes in the PPI network and play a leading role in regulation. M7G score was positively correlated with the expression of 6 m7G target genes (**Figure 6D**). The expression levels of six m7G target genes in the high m7G score group were significantly higher than those in the low m7G score group (**Figure 6E**). M7G target genes and m7Gscore were closely correlated with multiple immune checkpoint genes (**Figure 6F**). Through GSVA analysis, it was found that m7G target genes and m7Gscore were closely related to multiple carcinogenic pathways (**Figures 6G**, **H**).

### IHC and immunofluorescence results for FN1 and ITGB1

**Figure 7:**
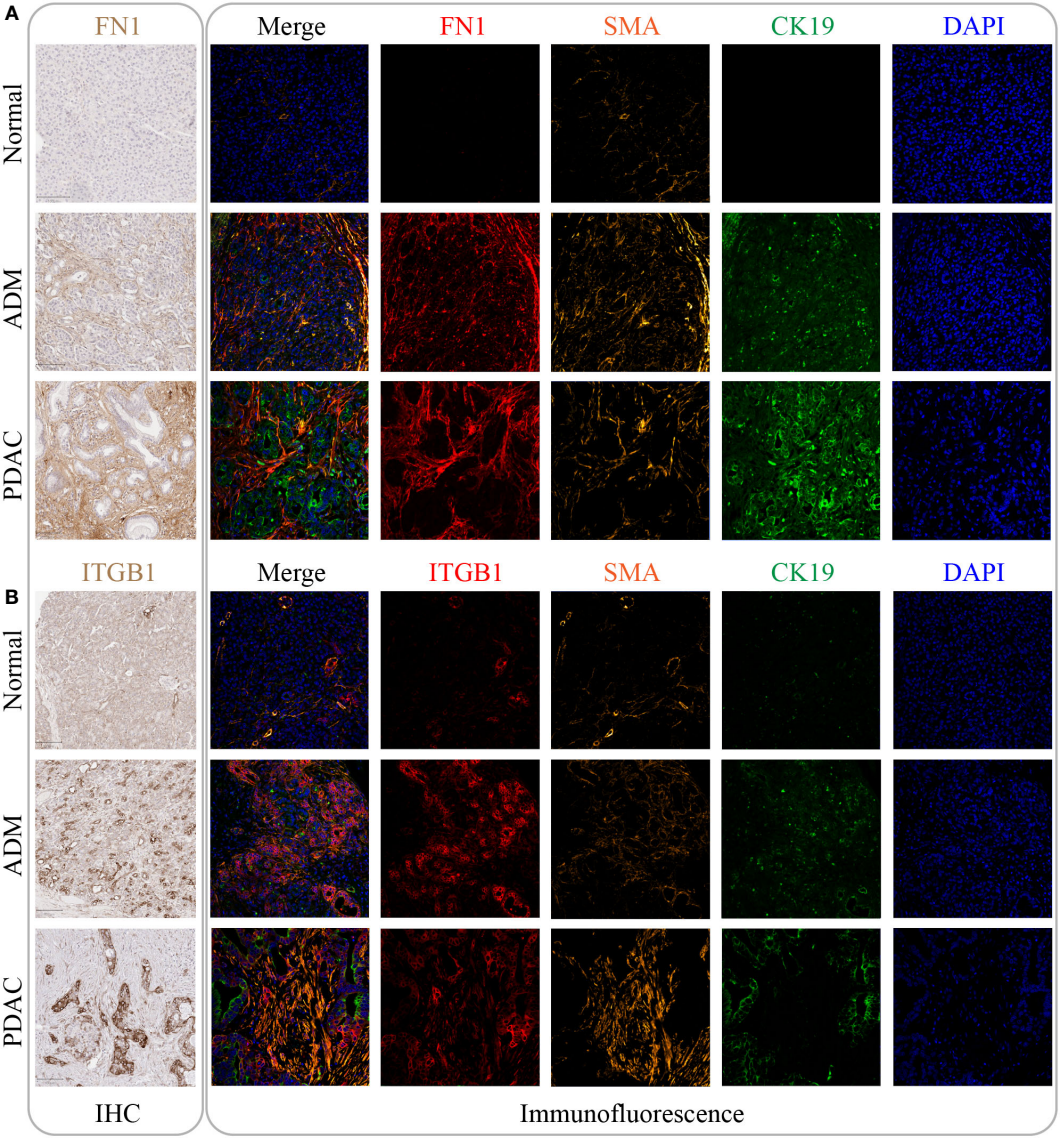
The expression of FN1 and ITGB1 in normal tissue, ADM tissue and PDAC tissue. **(A)** The expression of FN1 was gradually increased by IHC and immunofluorescence. Its expression location was mainly in the intercellular substance. **(B)** The expression of ITGB1 was gradually increased by IHC and immunofluorescence. Its expression location was mainly inside the cancer cell.

To validate the RNA results at protein level, we performed IHC and immunofluorescence analysis of the core genes (FN1, ITGB1) in the m7G methylation model (**Figures 7A**, **B**). Compared with the negative staining in normal pancreas acini, FN1 showed strong expression in the stroma of ADM and PDAC (**Figure 7A**), whereas ITGB1 showed strong expression in the epithelial cells of both ADM and PDAC (**Figure 7B**). The ADM epithelium was further validated by co-staining with the ductal marker CK19 and SMA highlighted activated stromal myofibroblasts and smooth muscle in vessel walls.

### Analysis of FN1 in pan-cancer

**Figure 8:**
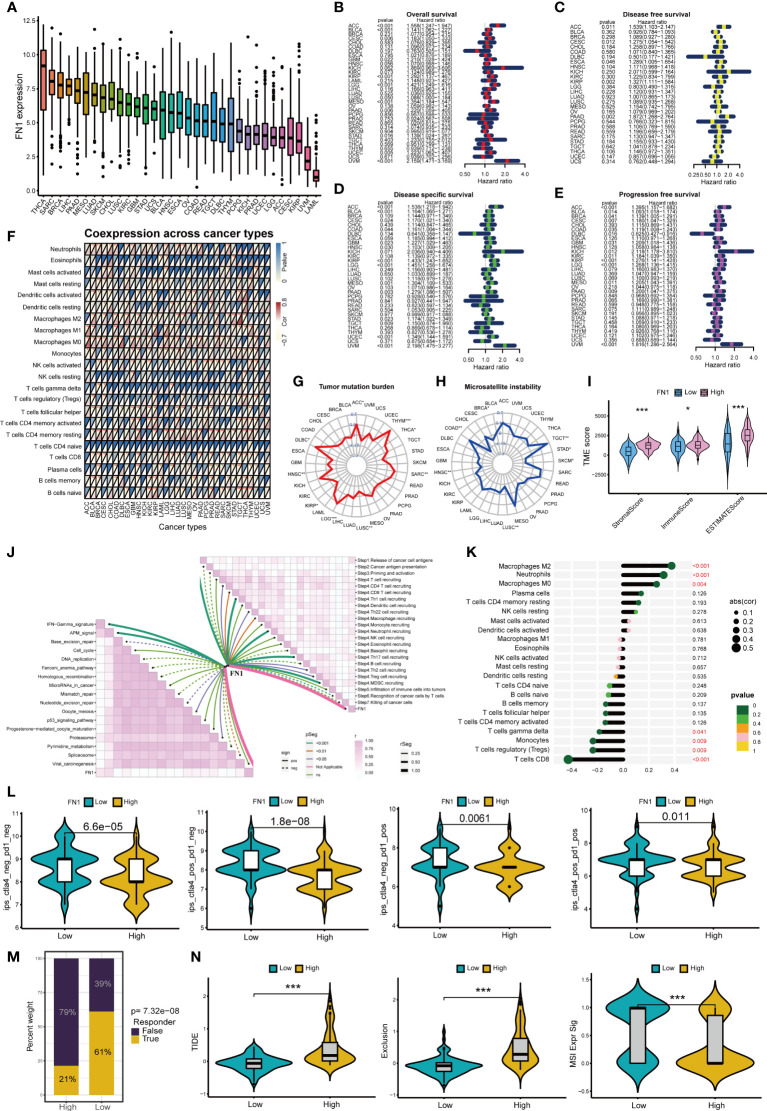
Analysis of FN1 in cancers. *p<0.05; **p<0.01; ***p<0.001. **(A)** Expression of FN1 in 33 cancers. **(B)** Overall survival of FN1 in pan-cancer. **(C)** Disease free survival of FN1 in pan-cancer. **(D)** Disease special survival of FN1 in pan-cancer. **(E)** Progression free survival of FN1 in pan-cancer. **(F)** Co-expression analysis of FN1 and immune cells in pan-cancer. **(G)** Tumor mutation burden of FN1 in pan-cancer. **(H)** Microsatellite instability of FN1 in pan-cancer. **(H) (I)** ESTIMATE analysis of FN1 high- and low-expression group. **(J)** Correlation between FN1, immunotherapy pathway and cancer immunity cycle. **(K)** Correlation analysis between FN1 and CIBERSORT immune cells. **(L)** Response rate of ICIs in FN1 high- and low-expression group. **(M)** Response of immunotherapy treatment in FN1 high- and low-expression group. **(N)** TIDE analysis of FN1 high- and low-expression group.

**Figure 8A** shows the expression of FN1 in 33 cancers, among which FN1 most expressed in THCA. **Figures 8B–E** shows that FN1 can affect overall survival、disease free survival disease specific survival and progression free survival of patients in a variety of cancers (including PAAD). Moreover, FN1 can affect immune cell infiltration in pan-cancer (**Figure 8F**). FN1 is closely associated with tumor mutation burden and microsatellite instability in a variety of tumors (**Figures 8G, H**). We further analyzed the role of FN1 in the immune microenvironment of pancreatic cancer. Based on the ESTIMATE analysis, we found that the estimate score, stromal score and immune score of FN1 high-expression group were significantly higher than those of FN1 low-expression group (**Figure 8I**). FN1 is also closely associated with multiple immunotherapy pathways and classical process of the cancer immunity cycle (**Figure 8J**). Using CIBERSORT algorithm, we calculated the correlation between FN1 and immune cells (**Figure S3A**). The results showed that FN1 was positively correlated with macrophages and neutrophils, and negatively correlated with various immune T cells (**Figure 8K**). **Figure 8L** showed that the response rate of PD1 and CTLA4 treatment in FN1 low-expression group was significantly higher than that in FN1 high-expression group, which was consistent with the results of m7G score model on the response rate of ICIs. We also calculated the response possibility of FN1 to immunotherapy by TIDE algorithm, and the results showed that the response of FN1 low-expression group was significantly higher than that of FN1 high-expression group (**Figure 8M**). Furthermore, the expression of TIDE score, exclusion score and MSI expr sig in the FN1 high-expression group was higher than that in low-expression group (**Figure 8N**). We analyzed the correlation of checkpoint genes between the high and low expressed FN1 groups, the result showed that multiple checkpoint genes were found to be highly expressed in the high-expression FN1 group (**Figure S3B**). These results were consistent with the results of m7G score model, suggesting that FN1 may cause immunotherapy resistance in pancreatic cancer patients through immune evasion.

### Analysis of ITGB1 in pan-cancer

**Figure 9:**
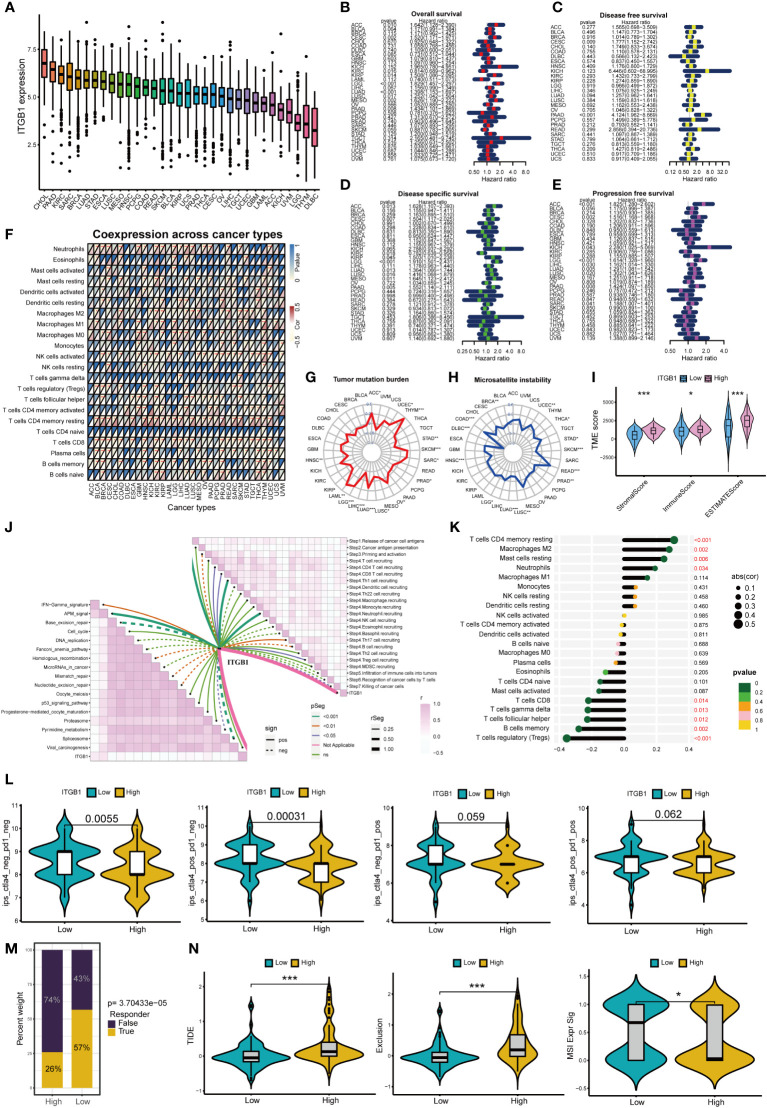
Analysis of ITGB1 in cancers *p<0.05; **p<0.01; ***p<0.001. **(A)** Expression of ITGB1 in 33 cancers. **(B)** Overall survival of ITGB1 in pan-cancer. **(C)** Disease free survival of ITGB1 in pan-cancer. **(D)** Disease special survival of ITGB1 in pan-cancer. **(E)** Progression free survival of ITGB1 in pan-cancer. **(F)** Co-expression analysis of ITGB1 and immune cells in pan-cancer. **(G)** Tumor mutation burden of ITGB1 in pan-cancer. **(H)** Microsatellite instability of ITGB1 in pan-cancer. **(I)** ESTIMATE analysis of ITGB1 high- and low-expression group. **(J)** Correlation between ITGB1, immunotherapy pathway and cancer immunity cycle. **(K)** Correlation analysis between ITGB1 and CIBERSORT immune cells. **(L)** Response rate of ICIs in ITGB1 high- and low-expression group. **(M)** Response of immunotherapy treatment in ITGB1 high- and low-expression group. **(N)** TIDE analysis of ITGB1 high- and low-expression group.

**Figure 9A** shows the expression of ITGB1 in 33 cancers, among which ITGB1 expressed highest in CHOL and PAAD. **Figures 9B–E** shows that ITGB1 can affect overall survival、disease free survival disease specific survival and progression free survival of patients in a variety of cancers (including PAAD). Moreover, ITGB1 can affect immune cell infiltration in pan-cancer (**Figure 9F**). ITGB1 is closely associated with tumor mutation burden and microsatellite instability in a variety of tumors (**Figures 9G, H**). ITGB1 can also affect the immune microenvironment in pancreatic cancer (**Figure 9I**). Based on the ESTIMATE analysis, we found that the estimate score, stromal score and immune score of ITGB1 high-expression group were significantly higher than those of ITGB1 low-expression group. ITGB1 is also closely associated with multiple immunotherapy pathways and classical process of the cancer immunity cycle (**Figure 9J**). Using CIBERSORT algorithm, we calculated the correlation between ITGB1 and immune cells (**Figure S3C**). The results showed that ITGB1 was positively correlated with macrophages and neutrophils, and negatively correlated with various immune T cells (**Figure 9K**). **Figure 9L** showed that the response rate of CTLA4 treatment in ITGB1 low-expression group was significantly higher than that of ITGB1 high-expression group, while there was no significant statistical difference to PD1 treatment between ITGB1 high- and low-expression group. We also calculated the response possibility of ITGB1 to immunotherapy by TIDE algorithm, and the results showed that the response of ITGB1 low-expression group was significantly higher than that of ITGB1 high-expression group (**Figure 9M**). In addition, the expression of TIDE score, exclusion score and MSI expr sig in the ITGB1 high-expression group was higher than that in low-expression group (**Figure 9N**). We analyzed the correlation of checkpoint genes between the high and low expressed ITGB1 groups, the result showed that multiple checkpoint genes were found to be highly expressed in the high-expression ITGB1 group (**Figure S3D**).

## Discussion

Although ADM is a benign and reversible process in the setting of acute pancreatitis, long-term pancreas inflammatory stimulation can lock metaplastic cells into a duct-like state. Persistent ADM has been proven to be a precursor lesion for the development of PDAC (28, 29). Pancreatic cancer develops through a series of genetic events triggered by different driver gene mutations. These differences in the mutated driver genes lead to differences in the molecular phenotypes and biological behaviors of pancreatic cancer, ultimately resulting in different clinical outcomes (30). Studies have shown that the progression from ADM to PDAC is driven by complex malignant bridging genes and pathways (31). It is still unclear whether these bridging genes can continue to play a malignant driving role and how they function after the occurrence of PDAC. As a newly-developed spatial genomics technology, DSP can accurately detect the *in situ* expression of RNAs and proteins in both ADM and PDAC areas simultaneously. This technology avoids contamination during the process of laser microdissection and RNA preparation (32). In this study, we collected tissues from 8 PDAC patients with a history of chronic pancreatitis, and with histological manifestation of the malignant progression from ADM to PDAC on the same tissue section. Analysis using DSP technology on these human PDAC tissue samples identified 224 bridging genes in the progression from ADM to PDAC. Among these 224 genes, there was a significantly a higher degree of overlap with the m7G methylation genes. Therefore, we speculated that the development of pancreatic cancer might be closely related to the m7G methylation genes.

Cluster analysis is a powerful tool that can classify tumors into subtypes based on their genomic similarities and differences in association with patients’ clinical parameters and outcome data. It can also facilitate comparative study of different subtypes and discovery of new tumor subtypes (33). Cluster 2 had the lowest overall survival rate, and Cluster3 had the highest overall survival rate. The expression of most m7G methylation genes in Cluster2 was significantly higher than that in cluster 3. Some classic oncopathways have also been significantly activated in Cluster2, such as TGF-beta, ERBB, Wnt and so on. These results suggest that m7G regulators may affect the malignant progression of pancreatic cancer through a variety of cancer pathways.

Malignant progression of tumor-related diseases is often accompanied by changes in cell morphology, such as ADM and epithelial to mesenchymal transition (34). Roland et al. found that PDAC is a process characterized by the extreme involvement of the ECM, and the changes in the ECM-receptor interaction pathway in PDAC are consistent with ECM remodeling (35). Functional analysis showed the most malignant Cluster2 was closely related to differentiation-related pathways, such as regulation of cell morphogenesis involved in differentiation. In this study, we found that several ECM and tumor microenvironment pathways were activated in cluster 2, such as ECM receptor interaction and cell substrate junction.

PDAC is characterized histologically by the presence of abundant desmoplastic stroma containing very small number of infiltrating lymphocytes, indicating an overall immunosuppressive microenvironment (36). It is well known that the degree of immune cell infiltration is closely related to the efficacy of immunotherapy and the prognosis of cancer patients. Studies have found that immune cell infiltration was regulated by a variety of epigenetic factors, including m6a and m5c methylation (37). But as a newer type of methylation, there are few immune-related studies on m7G methylation. The results of this study suggest that m7G methylation regulators may affect immune cell infiltration, which further affect the immunotherapy response and patient’s prognosis.

In this study, the PCA algorithm showed that m7G scores were negatively correlated with the overall survival rate. The Sankey chart showed that most of the cases in Cluster2 with the worst prognosis belong to the high m7G score group. These results support the notion that m7G regulators play an important role in the malignant progression of pancreatic cancer.

Through the TIDE score, we can intuitively understand the immune escape mechanism of the high and low m7G score groups. Studies have shown that in some tumors, although the degree of cytotoxic T cell infiltration is high, these T cells are often in a state of dysfunction. In other tumors, immunosuppressive factors can eliminate T cells infiltrating the tumor tissue (38). The TIDE score results showed that the immune escape mechanism of the low m7G score group was mainly by dysfunction, while the high m7G score group was mainly by immune exclusion.

In a phase I clinical trial of 207 patients with different types of advanced cancer who received ICI monotherapy, Brahmer et al. found that drug efficacy was relatively poor in patients with advanced pancreatic cancer (39). Another randomized phase II trial of 65 patients with metastatic pancreatic cancer who failed first-line treatment with 5-FU or gemcitabine showed that the disease control rate of the combined drug treatment was significantly better than that of the monotherapy (40). We performed immune checkpoint assessments of the different m7G scores groups. The results showed that tumors with low m7G scores had a higher rate of response to ICI monotherapy. ICI monotherapy had poor efficacy in the high m7G score group, although the efficacy of combination therapy was relatively good, which is consistent with the results of multiple clinical studies. Studies have shown that the tumor microenvironment contributes to ICI resistance. A nonimmunogenic tumor microenvironment could potentially inhibit the immune response and prevent the accumulation of immune lymphocytes in tumor tissues (41), thereby affecting the efficacy of ICI treatment and leading to the development of drug resistance. On the other hand, long-term pancreatic cancer survivors have high-quality neoantigens in the tumor microenvironment. Therefore, it is conceivable that targeting these neoantigens may improve the effectiveness of ICIs in the treatment of pancreatic cancer (42).

We constructed a PPI network for the 6 m7G target genes. In the PPI network, the core genes were FN1 and ITGB1. FN1 is a glycoprotein that is mainly involved in the processes of cell adhesion and migration. Studies have reported that FN1 expression is upregulated in a variety of tumors and is negatively correlated with patients’ prognosis. FN1 overexpression can be used as a molecular marker for the invasive phenotype of PDAC (43). Tsukamoto et al. found that alcohol consumption could induce pancreatitis in mice, increase FN1 expression and promote PDAC carcinogenesis (44). In a TGF-β treatment-induced PDAC model, Yuzuru et al. found that upregulation of FN1 was a hallmark of the ductal growth of PDAC (45). It has been shown that stromal cells are capable of inducing epithelial-mesenchymal transformation, an event that is closely associated with the progression of inflammation to tumors (46).Margareta et al. demonstrated that FN1 functions in epithelial misplacement (AEM) and adenomas with early carcinoma (AEC) transformation in colon cancer (47), suggesting that FN1 plays a role in the inflammatory transformation of cells to colon cancer. Proteomics study showed that abundant FN1 is present in extracellular vesicles (EVs) of PDAC and that high expression of FN1 reduces the sensitivity of PDAC to gemcitabine treatment (48). A member of the integrin family, ITGB1 was also reported to play an important role in PDAC carcinogenesis and biological behavior. ITGB1 signaling has been shown to promote the proliferation and metastatic ability of pancreatic carcinoma *in situ* in mice by stimulating the production of inflammatory cytokines (49). ITGB1 also influences the malignant progression of epithelioid-like ovarian cancer by regulating the production of the inflammatory factors IL-6, TGF-β1 and SDF-1 (50). A study by Oklahoma University suggested that ZIP4 could increase the resistance of pancreatic cancer patients to gemcitabine by upregulating the expression of ITGB1, which was associated with a poor prognosis (51). Another study from MD Anderson Cancer Center showed that GAL3 regulates the production of inflammatory cytokines in ITGB1. Inhibition of this pathway can reduce the growth and metastasis of pancreatic cancer in mice (49). These literature data and our PPI analysis results all suggest that the group of FN1 and ITGB1 genes interact with one another and exert their functions as a coordinated network. In this study, both immunohistochemical and immunofluorescence analysis showed that (1) FN1 protein was highly expressed in the stroma of ADM and PDAC lesions, and (2) ITGB1 protein was highly expressed in the epithelium of ADM and PDAC. This result suggests that increased expression of FN1 and ITGB1 is associated with the metaplastic transdifferentiation of normal pancreatic acinar cells to ductal cells through ADM and eventually, the development of PDAC.

FN1 and ITGB1 not only play a role in pancreatic cancer, but also be closely associated with overall survival, immune cell infiltration, tumor mutation burden and microsatellite instability in pan-cancer. This suggests that m7G score model and m7G target genes may be independent prognostic factors for a variety of tumors. The expression of FN1 and ITGB1 was positively correlated with macrophages and neutrophils, and negatively correlated with immune-related T cells. Studies have shown that tumor-associated macrophages (TAM) play an important role in tumor immune evasion (52). Various mediators in the tumor microenvironment mediate the recruitment of myeloid-derived suppressor cells (MDSC) and monocytes, and polarize macrophages through different signaling pathways, thereby promoting the formation of the immunosuppressive myeloid microenvironment. Meanwhile, tumor-associated neutrophils (TAN) are also an important part of the immunosuppressive myeloid microenvironment (53). Neutrophils in tumor microenvironment can inhibit the immune function of T cells, which leads to the failure of ICIs treatment (54). Both CTLA4 and PD1 ICIs treatment can activate immune checkpoint molecules expressed on the surface of T cells, thereby reactivating T cells to play anti-tumor role (55, 56). When T cells are depleted, tumors are more likely to form immunosuppressive microenvironments that help tumor cells evade immune surveillance (57). Moreover, the higher expression levels of FN1 and ITGB1, the lower response rate of patients to ICIs treatment. TIDE score, exclusion score and MSI score in high-expression group were also significantly higher than those in low-expression group. This also indicates that the high-expression group is more prone to immune evasion. Therefore, we conclude that FN1 and ITGB1 can lead to immune evasion in pancreatic cancer and reduce the response rate of ICIs by up-regulating the activity of macrophages and neutrophils, and down-regulating expression of immune T cells.

In summary, we used spatial genomics technology DSP to identify the bridging genes in the transition from normal parenchyma to ADM to PDAC. We found that these bridging genes highly overlapped with m7G methylation genes. The integrated model of ADM-Related m7G regulators was able to predict genomic instability, immune checkpoint treatment effectiveness, and overall survival in patients with pancreatic cancer. Once validated in large clinical trials, m7G score could be used to classify PDAC into different groups with different patterns of immune infiltration, genomic instability, and ICI response rate. M7G target genes have the potential to become novel diagnostic biomarkers or therapeutic targets of PDAC.

## Author contributors

HY: bioinformatics analysis, manuscript writing, and graphical visualization. JM-P: prepare tissue and coordinating the DSP study, conduct immunohistochemistry, and manuscript editing. AL: experimental design and supervision. AG: experimental supervision. J-lL: provided input to the conception and experimental design. YR: conducted the DSP study and preliminary data analysis. AN: performed initial bioinformatics analysis of the data. HN: bioinformatics analysis supervision. BG: conceptual design, bioinformatics analysis, manuscript revision, and providing fund. Z-hG: conceptual design, selection of experimental tissue, analyze histology and immunohistochemistry data, and manuscript revision. All authors contributed to the article and approved the submitted version.

## Data availability statement

The original contributions presented in the study are publicly available. This data can be found in the GEO database, accession number: GSE208536.

## Ethics Statement

This study was approved by the Institutional Research Ethics Board of the McGill University Health Center (MP-37-2018-4399, MP-37-2018-3171, MP-37-2020-5723). Written informed consent was waived by the Ethics board for this retrospective study using archived tissue only.

## Funding

This work was supported by the National Natural Science Foundation of China [Grant No. 81800573], Peking University People’s Hospital Research and Development Funds [Grant No. RDY2018-06 and RS2021-08] and Academic Rising Star Program of Peking University People’s Hospital [Grant No. RS2021-08].

## Conflict of Interest

The authors declare that the research was conducted in the absence of any commercial or financial relationships that could be construed as a potential conflict of interest.

## Publisher’s Note

All claims expressed in this article are solely those of the authors and do not necessarily represent those of their affiliated organizations, or those of the publisher, the editors and the reviewers. Any product that may be evaluated in this article, or claim that may be made by its manufacturer, is not guaranteed or endorsed by the publisher.

## Supplementary Material

Supplementary Figure 1 The GO and KEGG enrichment analysis of the three PDAC clusters. **(A–C)** GO enrichment analysis of the three clusters. **(D–F)** KEGG enrichment analysis of the three clusters. **(G)** The differentially expressed genes among the three types were intersected, resulting in a total of 907 genes. **(H, I)** GO enrichment analysis of the 907 genes. **(J, K)** KEGG enrichment analysis of the 907 genes. **(L)** Area under the curve (AUC) for time-dependent receiver operating characteristic curves of the m7G score model.

Supplementary Figure 2 The 25 drugs were selected for patients with low m7G score.

Supplementary Figure 3 CIBERSORT and checkpoint analysis of core genes. **(A)** CIBERSORT analysis of FN1.**(B)** checkpoint analysis of FN1. **(C)** CIBERSORT analysis of ITGB1. **(D)** checkpoint analysis of ITGB1.
