# Supplementary figures and images for: An integrated model of acinar to ductal metaplasia-related N7-methyladenosine regulators predicts prognosis and immunotherapy in pancreatic carcinoma based on digital spatial profiling
Source: Front Immunol. 2022 Jul 28;13:961457. doi: 10.3389/fimmu.2022.961457 (PMC9377277; doi:10.3389/fimmu.2022.961457)

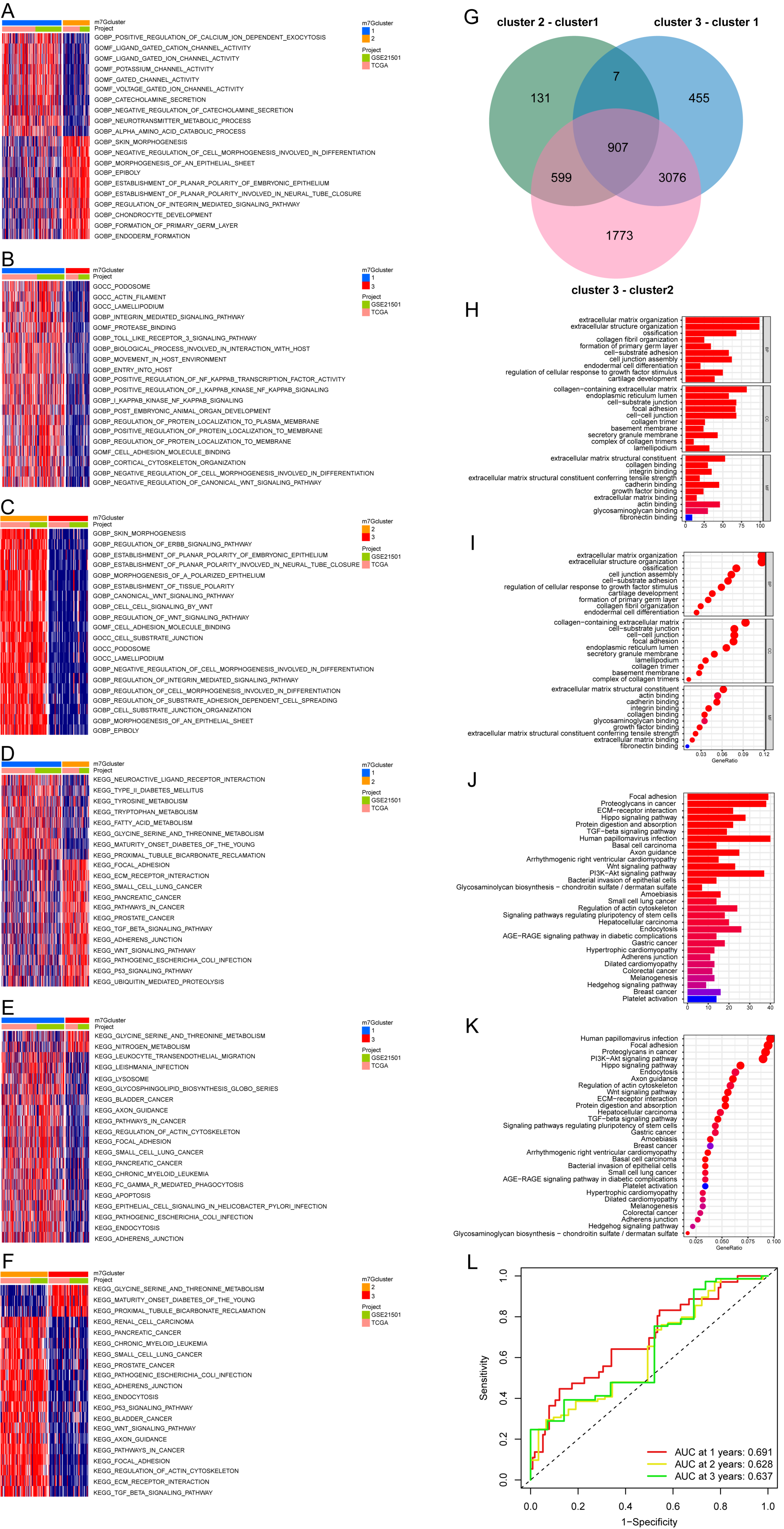

Supplement: Supplementary Figure 1 — The GO and KEGG enrichment analysis of the three PDAC clusters. (A–C) GO enrichment analysis of the three clusters. (D–F) KEGG enrichment analysis of the three clusters. (G) The differentially expressed genes among the three types were intersected, resulting in a total of 907 genes. (H, I) GO enrichment analysis of the 907 genes. (J, K) KEGG enrichment analysis of the 907 genes. (L) Area under the curve (AUC) for time-dependent receiver operating characteristic curves of the m7G score model. [file Image_1.tif]

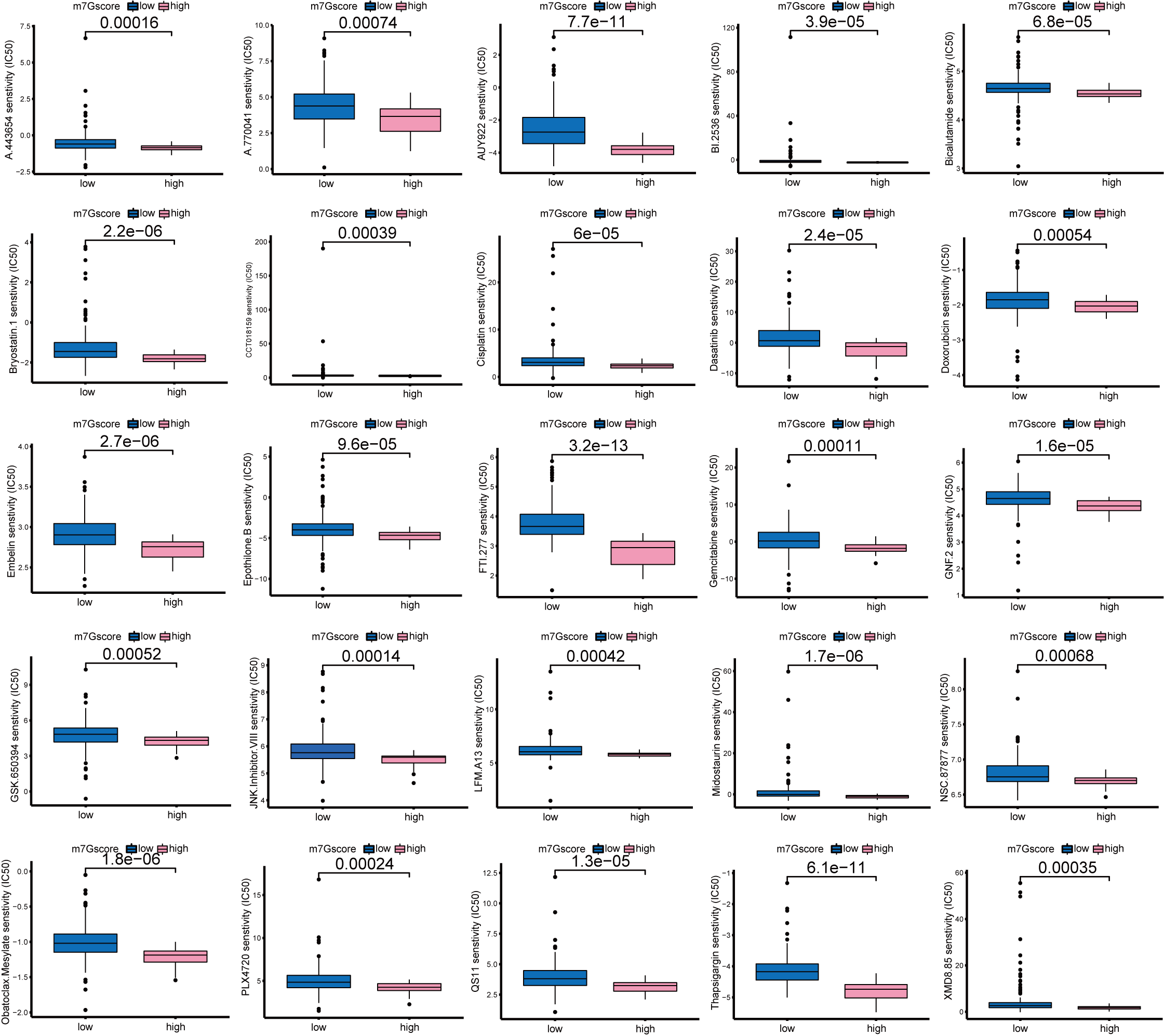

Supplement: Supplementary Figure 2 — The 25 drugs were selected for patients with low m7G score. [file Image_2.tif]

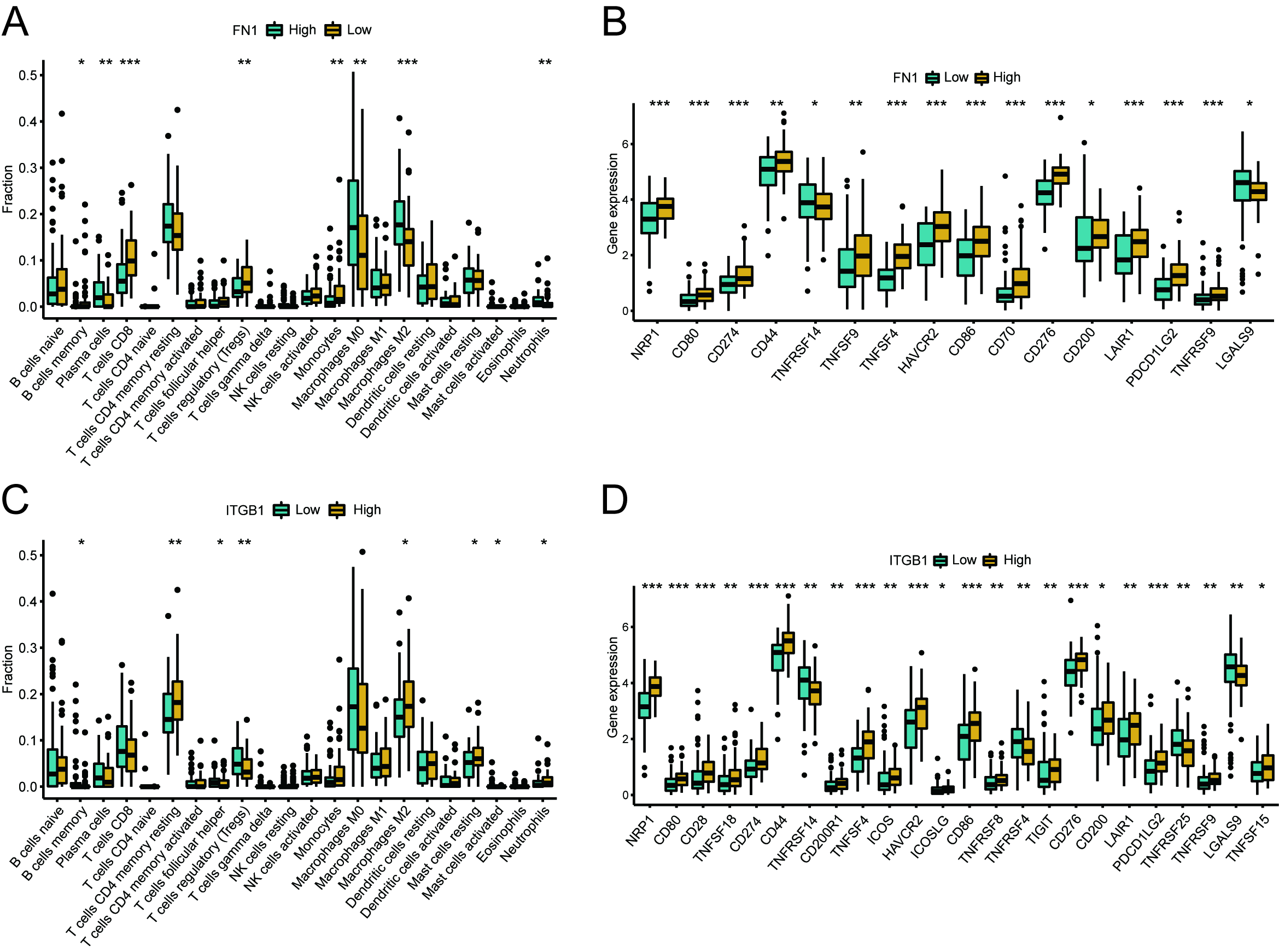

Supplement: Supplementary Figure 3 — CIBERSORT and checkpoint analysis of core genes. (A) CIBERSORT analysis of FN1.(B) checkpoint analysis of FN1. (C) CIBERSORT analysis of ITGB1. (D) checkpoint analysis of ITGB1. [file Image_3.tif]
